# Interpreting 340B contract pharmacy growth: who really benefits?

**DOI:** 10.1093/haschl/qxad076

**Published:** 2023-12-09

**Authors:** T Joseph Mattingly

**Affiliations:** Department of Pharmacotherapy, University of Utah College of Pharmacy, Salt Lake City, UT 84112, United States

**Keywords:** 340B, contract pharmacy

In this issue of *Health Affairs Scholar*, McGlave et al^1^ do a remarkable job linking the Health Resources and Services Administration's Office of Pharmacy Affairs (OPA) database to National Council for Prescription Drug Program (NCPDP) data to evaluate trends in 340B contract pharmacy participation. While the data demonstrate that 340B contract pharmacy growth has been predominantly realized by the 4 largest retail pharmacy chains, these findings can be viewed differently based one's interpretive filter^2^ reflecting either a positive or negative outcome when the truth may be more nuanced.

First, 340B was designed to benefit the covered entity, not the contract pharmacy,^3^ so the characteristics of participating pharmacies is less relevant to the evaluation of 340B. The evolution of the 32-year-old 340B program has created several incentives, many of which have been controversial, which include hospital profitability,^4,5^ hospital-physician consolidation,^6^ pharmacy service offerings,^7^ and brand-generic dispensing differences.^8^

Second, references to the location of contract pharmacies and implying that there should be concern if contract pharmacies are located “in areas with fewer uninsured and more wealthy patients” may be missing a very important point.^1^ County-level analyses of contract pharmacy locations may distract from the ultimate question of “who benefits” from the 340B program.^9^ In a contract between a covered entity and nonaffiliated pharmacy, the covered entity captures a proportion of the revenue associated with the prescription dispensation at that pharmacy, regardless of pharmacy ownership characteristics or physical location (Figure 1). Without the 340B arrangement, a patient could simply fill that prescription at the same pharmacy and the covered entity would earn no revenue. Patients living in high-income, heavily resourced counties or zip codes traveling to a covered entity in a low-income, underresourced county to receive care and then receiving prescriptions at pharmacies near their homes ultimately benefit both the contract pharmacy and the covered entity. This could be considered a transfer of dollars typically spent in a wealthy area to a lower-income area. If the covered entity reinvests those dollars in care services, then this should have positive implications for patients living in the low-income, underresourced county and help us improve health equity.

**Figure 1.**
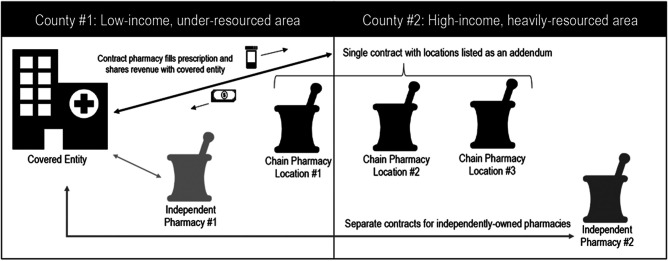
340B Covered entity and pharmacy contracting example.

Third, the issue of covered entities contracting with “chain-owned” vs “independently owned” pharmacies may be further complicated by operational and administrative factors the covered entity must consider. When the Affordable Care Act encouraged 340B expansion by allowing covered entities to contract with multiple pharmacies,^10^ leaders within these covered entities (many of which are nonprofit hospitals) were given an opportunity to establish relationships with nonaffiliated pharmacies to provide dispensing services that now qualified under the 340B drug discount program. The key word in a contract pharmacy relationship, from an implementation standpoint, is *contract*. This requires legal support and may be administratively burdensome for many large nonprofit hospitals. So, when given a discrete choice to establish a contract between a large retail chain with 20 locations surrounding a hospital or to establish 20 different contracts with 20 independent pharmacies in the same area, a hospital administrator is making an efficient decision to establish 1 contract with a pharmacy chain.

Similarly, ongoing maintenance of the contractual relationship requires the covered entity to function like a managed care organization with a “pharmacy network” that steers patients to contract pharmacies and may have some accountability to the quality of services provided. To the authors' point, an analysis of US pharmacy closures from 2009 to 2015 demonstrated that independent pharmacies and pharmacies in urban areas serving disproportionately low-income populations were at an increased risk of closure.^11^ From the covered entity perspective, establishing new contracts with pharmacies at a higher risk of closure would not be prudent.

I agree with the authors that the “lower participation among pharmacies that are at higher risk of closure is concerning,” but incentivizing or specifying whether a covered entity contracts with a chain or an independent pharmacy may be more complicated as a policy solution and may not help meet the original goals of the 340B program. The authors do a great job explaining how the NCPDP data are organized, with the cutoff to be considered a “chain” is having 4 or more pharmacies under common ownership.^12^ For example, if we introduce policy that limits a covered entity from contracting with the NCPDP definition of a chain or just incentivizes more contracts with NCPDP-defined independent pharmacies, we will create a disincentive for a successful independent pharmacy owner from expanding to a fourth location (or just force that owner to incorporate his fourth location under a new legal structure).

Finally, McGlave et al conclude that “pharmacy chains have benefited from the expansion of the 340B program, raising concerns about the feasibility of participation from independent pharmacies” without the appropriate context of the intent of the 340B program and without assessment of all potential stakeholders who benefit from the 340B program. With that said, McGlave et al should be commended for pointing out that a potential unintended consequence associated with the implementation of the 340B program is an economic environment that favors a “covered entity-chain pharmacy” arrangement over relationships with smaller, independently owned businesses. Policy makers should revisit the intent of the 340B program and conduct a full assessment that considers whether the original policy goals have been achieved and whether achieving those goals outweighs potential distortions in the outpatient pharmacy market.

## Supplementary Material

qxad076_Supplementary_Data

## References

[1] McGlave C, Bruno JP, Watts E, Nikpay S. 340B Contract pharmacy growth by pharmacy ownership: 2009–2022. Health Aff. 2023. 10.1093/haschl/qxad075 PMC10985927 38756399

[2] Druckman D, Olekalns M, Smith PL. Interpretive filters: social cognition and the impact of turning points in negotiation. Negot J. 2009;25(1):13–40. 10.1111/j.1571-9979.2008.00206.x

[3] Fein AJ. Challenges for managed care from 340b contract pharmacies. J Manag Care Spec Pharm. 2016;22(3):197–203. 27003548 10.18553/jmcp.2016.22.3.197 PMC10398234

[4] Conti RM, Bach PB. The 340B drug discount program: hospitals generate profits by expanding to reach more affluent communities. Health Aff. 2014;33(10):1786–1792. 10.1377/hlthaff.2014.0540 PMC4591849 25288423

[5] Conti RM, Nikpay SS, Buntin MB. Revenues and profits from Medicare patients in hospitals participating in the 340b drug discount program, 2013-2016. JAMA Network Open. 2019;2(10):e1914141. 31664442 10.1001/jamanetworkopen.2019.14141 PMC6824218

[6] Desai S, McWilliams JM. Consequences of the 340B drug pricing program. N Engl J Med. 2018;378(6):539–548. 29365282 10.1056/NEJMsa1706475 PMC6073067

[7] Wu T, Williams C, Vranek K, Mattingly TJ. Using 340B drug discounts to provide a financially sustainable medication discharge service. Res Soc Adm Pharm. 2019;15(1):114–116. 10.1016/j.sapharm.2018.03.065 29606609

[8] Clark BL, Hou J, Chou CH, Huang ES, Conti R. The 340B discount program: outpatient prescription dispensing patterns through contract pharmacies in 2012. Health Aff. 2014;33(11):2012–2017. 10.1377/hlthaff.2014.0833 PMC4545491 25367997

[9] Nikpay S, Gracia G, Geressu H, Conti R. Association of 340B contract pharmacy growth with county-level characteristics. Am J Manag Care. 2022;28(3):133–136. 35404549 10.37765/ajmc.2022.88840

[10] Kishore S, Nayak RK, Kesselheim AS. 340B—Where do we go from here? JAMA. 2023;330(7):593–594. 10.1001/jama.2023.11056 37505512

[11] Guadamuz JS, Alexander GC, Zenk SN, Qato DM. Assessment of pharmacy closures in the United States from 2009 through 2015. JAMA Intern Med. 2020;180(1):157–160. 31633745 10.1001/jamainternmed.2019.4588 PMC6806432

[12] National Council for Prescription Drug Programs. NCPDP dataQ™ Pharmacy Database File Standard Implementation Guide Version 3.1. Updated May 2023. Accessed October 23, 2023. https://dataq.ncpdp.org/

